# The first year of COVID-19 vaccine roll-out in Africa: challenges and lessons learned

**DOI:** 10.11604/pamj.supp.2022.41.2.33686

**Published:** 2022-03-29

**Authors:** Balcha Masresha, Miguel Angel Sanchez Ruiz, Phionah Atuhebwe, Richard Mihigo

**Affiliations:** 1 World Health Organisation, African Regional Office, Brazzaville, Congo,; 2 ECDC Fellowship Programme, Field Epidemiology path (EPIET), European Centre for Disease Prevention and Control (ECDC), Solna, Stockholm, Sweden,; 3 Santé Publique France, Provence-Alpes-Côte d'Azur, France

**Keywords:** COVID-19, COVID-19 vaccination, Africa, pandemic response, pandemic prepared

## Abstract

**Introduction:**

in the first year following the introduction of COVID-19 vaccines, only 6.8% of the total population in the 47 countries in the WHO African Region have received full vaccination. In an emergency context, the intra-action review helps countries to assess their progress and document what has worked and not worked.

**Methods:**

we reviewed and identified the key lessons and challenges documented in the reports from intra-action review of COVID vaccine roll out in 22 African countries.

**Results:**

all countries documented high level political commitment, but a serious shortage of COVID-19 vaccines and funding. Seven countries identified gaps in microplanning because of lack of funding or due to the unpredictability in the type and volume of vaccine supplies. The shortage of operational funding also affected training of health workers and hampered the expansion of service delivery. The countries implemented multi-channel communications and social mobilisation activities, alongside social media engagement and social listening. However, country capacity was limited in terms of timely responding to infodemics. Hesitancy among health workers and the general population was a challenge in most of the countries.

**Conclusion:**

countries have gained valuable experiences exploring various COVID-19 vaccination delivery models, including implementing the integration of COVID-19 vaccination within routine health care programs. There is a need to regularly monitor or do studies measuring public perceptions towards COVID-19 vaccination in order to drive the demand generation efforts, as well as use evidence in addressing hesitancy.

## Introduction

The SARS-COV-2 virus and coronavirus disease 2019 (COVID-19) were first identified in December 2019, and spread rapidly across the globe. On 11^th^ March 2020, the World Health Organization (WHO) declared the COVID-19 outbreak a global pandemic [1]. By the end of December 2021, countries in the WHO African region have reported 7,262,708 confirmed COVID cases and 156,290 deaths [2].

Various public health measures are being implemented since the start of the pandemic, and the development of vaccines has been progressing very fast. As of 31 December 2021, a total of 197 types of vaccines are under clinical development and 134 are in pre-clinical stages [3]. WHO has granted Emergency Use Listing (EUL) for 10 vaccines as of 31 December 2021 [4]. The evolution of the COVID-19 pandemic in the coming year will heavily depend on the progress of national and global vaccination programs. Most countries developed their National COVID-19 Vaccine Deployment Plans (NDVPs) by February 2021, in preparation for the arrival of vaccines. As per the global planning guidance, the national plans outlined the target populations and resources required for phased vaccination, based on the proposed priority setting, and the operational approaches countries select to deliver services to the target population [5].

Several initiatives aimed at providing equitable access to COVID-19 vaccines have been launched in the past 18 months. In the initial months of vaccine introduction, eligible countries were mostly sourcing their vaccines from the COVAX facility, while many countries (including non-GAVI eligible countries) organised a direct purchase or received vaccines from other countries through bilateral negotiations and donations [6]. Unequal access to vaccines is one of the main challenges to tackle the pandemic; with low vaccination coverage rates in most low-to-middle-income countries where COVID-19 continues to be a major concern [7]. By the end of 2021, a total of 179,289, 703 doses of COVID vaccines have been administered in 47 countries in the African Region with 76,162,804 persons fully vaccinated (6.8% of the total population in the Region) [8].

As the number and types of vaccines granted WHO Emergency Use Listing (EUL) grows, and as the supply of vaccines to countries in the African Region improves, addressing service delivery issues continues to require intensive efforts at different levels; including regulation and registration of vaccines, operational readiness, supply chain and logistics, surveillance data and monitoring, vaccine safety and surveillance for adverse event following immunisation (AEFI), and risk communication [9].

In the months from April to December 2021, 26 countries in the WHO African Region conducted desk reviews of the progress with the introduction of COVID-19 vaccines. These multi-stakeholder desk reviews, called Intra-Action Reviews (IARs) were conducted using a standard protocol, reviewing processes and outcomes across various thematic areas including: planning and coordination; regulation and vaccine safety; training and deployment of human resources; service delivery; risk communications and community engagement; monitoring and data analytics [10]. The protocol involves using trigger questions crafted to help drill down into the issues in each of the thematic areas. The objective of IARs is to help countries learn early lessons, identify bottlenecks to implementation and generate action points to improve program performance.

This study discusses the challenges of the COVID-19 vaccine roll-out and the lessons learned at different levels in African countries throughout 2021, according to the findings documented in the various intra-action review reports, with a view to help optimize the vaccination response in 2022.

## Methods

We reviewed the reports of 22 out of the 26 countries that have completed the intra-action review exercises to better understand what the major challenges and lessons were with regards to COVID-19 vaccine roll out. The intra-action review reports from São Tomé and Principe, the Republic of Congo, Madagascar and Zimbabwe were not available to review at the time of this write-up. Twenty-one out of the 22 IARs were conducted as desk review activities, while Kenya conducted a full-scale post-introduction evaluation, including field visits to selected counties and health facilities.

Two of the investigators went through the reports and codified the lessons and best practices according to thematic areas. The data were analysed thematically according to seven implementation domains: 1) planning and coordination; 2) training and human resources; 3) vaccine and cold chain logistics; 4) communication, demand generation and community engagement; 5) service delivery; 6) safety monitoring and surveillance for adverse events; 7) monitoring and data analytics.

Frequency statistics were used to describe programmatic findings and operational challenges among the countries in this series. We also reviewed the data related to doses of vaccine received, and administrative vaccination coverage for the 22 countries by the end of December 2021 to provide some context to the IAR findings. Coverage with at least one dose of COVID-19 vaccine was defined as the proportion of persons who received a first dose of any COVID-19 vaccine out of the total population. Full vaccination was defined as receipt of 2 doses of AstraZeneca, Sinopharm, SinoVac, Pfizer-BioNTech, Moderna, Sputnik V vaccine or 1 dose of Janssen COVID-19 vaccine.

## Results

All 22 intra-action review exercises were conducted between April and October 2021. As of the end of December 2021, these 22 countries have attained full COVID-19 vaccination coverage rates, ranging from 0.13% for DR Congo to 44% in Botswana. Only four countries in this group have attained full vaccination coverage rates above 10%. Less than 5% of the total population is fully vaccinated in 12 of the 22 countries. The principal findings, challenges and lessons learnt in these countries are described below.

### Planning and coordination

Fifteen (68%) countries indicated that there was adequate high-level political commitment and engagement in the respective countries to promote COVID-19 vaccination. In Ghana, the Inter-Ministerial Coordinating Committee is chaired by the President of the Republic. The national coordination of COVID vaccination was supported by the Inter-agency coordination committee (ICC) in 9 countries, with an expanded role to cover COVID vaccination beyond the usual terms of reference covered by the ICC. Weaknesses in the national level coordination of COVID vaccination was noted in 13 (59%) countries. These gaps were documented in the coordination of activities between the different arms of COVID response or among the health partners or between the health sector and other sectors. Eight countries reported that the regulatory approval of COVID-19 vaccines was processed in an expedited manner. Botswana, Guinea and Togo had some dedicated local funding for the purchase of COVID-19 vaccines or to support operational roll out.

All 22 countries developed their national COVID-19 vaccination plans in late 2020 - early 2021, well before the types and volumes of vaccines available to them as well as the timing of delivery were known. These plans were based on the global guidance, and using the prioritisation criteria proposed by the global Strategic Advisory Group of Experts (SAGE). The national plans were all developed with a view to phasing the service delivery starting with high priority grouping and proceeding to include other groups in a phased manner. Four countries identified that these national plans required updating by the time of the IAR exercise, considering the receipt of different types of vaccines, the vaccine consumption rates and expiry dates.

In the months until September 2021, the volume of vaccines available for countries was quite small; the delivery schedules and amounts were erratic. Sixteen (73%) of the 22 countries in this series outlined this shortage and erratic supply of vaccines as a major challenge for planning and implementation. Liberia reported that, due to the low volume of vaccine supplies, it was difficult to meet the demand for vaccines after the country launched intensive communication and demand generation activities. Uganda reported that it was particularly difficult to plan for a vaccination activity of unknown duration and with inadequate inputs. Introducing multiple types of new vaccines into national immunisation programs in a short period of time, and especially having vaccines with short shelf-lives was a significant operational challenge in 15 (68%) countries.

Seven countries identified gaps in microplanning because of lack of funding or due to the unpredictability in the type and volume of vaccine supplies. Another major challenge was the shortage or delayed disbursement of funds for the roll-out of vaccination activities, reported by 18 (82%) of the 22 countries.

### Training and human resources

Countries used existing immunisation program expertise and working systems to roll out COVID-19 vaccines. All countries reported that they leveraged the skills within the program and within the health sector in rolling out COVID-19 vaccines. However, there was a need for health worker training because of the peculiar characteristics of the new COVID-19 vaccine products, the target groups which differ from other vaccination services, and the phased approach. Fourteen (64%) countries reported that the training of health workers and provision of technical guidance was suboptimal. The trainings were mostly conducted online or done very briefly in order to avoid large and extended physical gatherings. Moreover, the recommended demonstration or drill sessions were not held. Simulation exercises were not systematically conducted at national and subnational level in any of the countries. These challenges were mostly related to insufficient or delayed disbursement of funding, and the hasty roll-out of vaccines once shipments started to arrive. In 4 countries, additional staff were recruited in order to meet the needs for service delivery. Ten countries identified inadequate supervision as a problem.

### Service delivery

Ten (45%) countries noted that they started service delivery in limited sites because of the shortage of vaccines and funding. Vaccination service was provided in static and outreach sites in all 22 countries, but mobile teams were implemented in 11 countries. In Sierra Leone, there were reports that mobile teams are not well trusted by the community, and are not often used. Service delivery was also affected by other factors. For example, many countries in Western Africa experienced declines in vaccine uptake during the month of Ramadan.

Three countries indicated that they implemented vaccination services in hard-to -reach populations (including refugees and displaced persons). Liberia provided vaccination to refugees along counties bordering Guinea, Ivory Coast and Sierra Leone, while Ethiopia extended services to internally displaced persons hosted in some provinces. The Democratic Republic of the Congo (DRC) set up vaccination sites in certain prisons and mining sites. All the countries stated that, revisions were made on the initial NDVP phasing to expand vaccination services to broader population groups after a few months of implementation. This was due to low COVID-19 vaccine uptake among the priority groups, and the risk of vaccine expiry since received vaccine shipments had a short shelf-life. A reduced rate of vaccination and/ or concerns about hesitancy among women were noted in Liberia, South Sudan, Sierra Leone, Cameroon and Chad.

All the countries recommended better integration with routine services, in terms of actual service delivery, social mobilisation and communication. Three countries reported that COVID-19 vaccination was negatively affecting routine immunisation services through the disruption of scheduled outreaches and static services. In the Gambia, vaccination teams were overwhelmed with work negatively affecting routine vaccination services, and the demand and uptake of human papillomavirus (HPV) vaccination was negatively affected by the COVID-19 vaccine introduction. In Kenya, nearly two thirds of the counties reported having routine immunization services and coverage affected by the introduction of the Covid-19 vaccines. Liberia reported challenges with balancing COVID-19 vaccination activities and routine immunization especially outreach services.

Zambia had very good integration of COVID vaccination services with TB, HIV antiretroviral treatment (ART) clinics and with family planning services. Sierra Leone recommended integrating COVID vaccination along with other interventions during periodic health days like child health days, those commemorating malaria control, tuberculosis control, and the annual African Vaccination week etc. Cameroon recommended that the strategy to increase access to COVID-19 vaccines should consider integrating vaccination within pre-existing health services, including chronic illness follow-up clinics.

### Communication, demand generation and community engagement

Twelve (55%) countries indicated that they implemented multichannel/multimedia communication and social mobilisation activities, while 9 countries introduced some sort of social listening and rumor tracking systems. All the countries included social media engagement as part of their communications strategies. However, many of the countries indicated weaknesses in their capacity to respond to the rumors in a timely manner. Botswana introduced a successful branded mobilisation campaign called “#ArmReady” just before the start of COVID-19 vaccination. Eleven countries set up toll-free telephone call centers for the provision of relevant information to the public. Nineteen (86%) countries indicated that weak implementation of communication and community engagement activities impacted on their coverage. Misinformation through the social media was mentioned as a major challenge in 13 countries. Hesitancy to take COVID vaccine was noted among health workers in 10 (45%) countries, and in other population groups in 7 countries.

Five countries indicated that there was inadequate knowledge of the behavioural drivers influencing vaccine hesitancy. In seven countries, some sort of survey was done to gather evidence on the public perception of COVID-19 vaccination and tailor their messaging accordingly. South Sudan implemented focus group discussions among members of various public groups, and a survey among health workers to understand perceptions and attitudes towards COVID vaccines. Ghana and Togo conducted studies on the knowledge, attitude and practices (KAP) regarding COVID-19 vaccines in order to adapt messages, while Cameroon explored vaccine acceptability among the population and recommended carrying out qualitative studies to understand the reasons for vaccine hesitancy in certain groups.

Uganda reported that the scarcity of vaccines was one factor that fuelled mistrust. In the Gambia, inadequate AEFI investigation and feedback, as well as conflicting messages on the target group were mentioned as important factors for hesitancy. In Eswatini, it was reported that an increased number of adverse events during the third wave may have contributed to vaccine hesitancy and reduced vaccine uptake.

In Kenya and Uganda, teachers and health care workers were identified as vaccine hesitant, while weak vaccine acceptance was also identified among health workers in Burkina Faso, Cameroon and Niger. The uptake of second doses was low in Liberia and Gambia due to stockout issues. In addition, Liberia mentioned AEFIs with the first dose vaccine as a reason for defaulting on the second dose. In Zambia, second dose vaccine was offered at specific sites only, different from the initially planned outreach/mobile sites. This was done as a result of low vaccine stocks and led to some hesitancy and high defaulter rate.

### Vaccine safety and AEFI surveillance

All the countries built their AEFI reporting on existing systems. However, digital tools were introduced for AEFI reporting following COVID-19 vaccination in 6 countries. AEFI reporting was considered very weak in 18 (82%) countries, with 8 (36%) countries identifying weak coordination between various parallel AEFI reporting systems or channels. Ethiopia and Uganda reported discrepancies in the numbers reported through the pharmacovigilance system and the AEFI surveillance reporting run though the immunisation program. In Kenya, one of the factors for weak health worker reporting of AEFIs was the fear of consequences from senior managers.

In 7 countries, multiple different channels were set up for AEFI reporting by health workers and the public, including passive reporting by clients using telephone hot lines and active reporting by health workers. The national AEFI committees, tasked with the responsibility of reviewing serious AEFI cases and doing the causality assessment and classification, were not functioning optimally, with subsequent delays in the causality assessment and feedback in 11 (50%) of the 22 countries. The shortage of AEFI kits was documented as a challenge in 8 (36%) countries. A few countries mentioned the challenge of media reporting of alleged AEFI cases ahead of any official investigation and communication. Such reporting was considered even more problematic in the case of severe AEFIs, when the media reports implicate the vaccines or the vaccination process, without being informed by the results of causality assessment by the national AEFI committees.

### Vaccine logistics and cold chain

Cold chain space was adequate in 14 (64%) of the 22 countries, mainly since the volume of vaccines available was limited throughout most of 2021. Eight countries had gaps in ultra-cold chain equipment for the storage of mRNA vaccines at national level. Sixteen (73%) countries reported the challenge of shortage of vehicles and/ or lack of funding for fuel to move vaccines and vaccinators outside of urban and district centers. In 4 countries, beyond the Ministry of Health, other stakeholders and partners (defence forces, UN agencies, NGOs or the private sector) assisted with moving vaccines and vaccination teams to subnational depots or to the district level. Ghana utilised drones to deliver more than 450,000 vaccines to 42 districts across the country by the time of the IAR.

Nine (41%) countries indicated that vaccine distribution was done efficiently across districts. Five countries implemented reverse logistics systems to collect and redistribute vaccines from districts with low utilisation rates to areas where there were bigger needs and utilisation rates. Senegal and Zambia rolled out the Logistimo software for stock management in a limited number of districts.

### Monitoring and data analytics

There was an attempt to introduce digital monitoring tools in most countries to track stocks and to monitor coverage. Eight countries implemented real-time digital monitoring systems to keep track of coverage and vaccine stocks. The DHIS2 Tracker platform was used for data capture and management in 7 countries. Three countries introduced verifiable vaccination records including QR codes. In addition, Ghana introduced holograms on the vaccination cards. Only Kenya and Eswatini reported having a defaulter tracing system or an automated second dose reminder system to ensure that persons who took their first dose return for their second shots at the right time.

The lack of clearly enumerated target population was documented as a problem for adequate coverage monitoring at the operational level in 4 countries. Nine countries did not have the adequate number of digital devices for use at all service delivery centers. In addition, the shortage of data entry clerks at the operational level was a challenge for timely data availability at the national level in 9 countries. Shortage of monitoring tools (various paper forms or electronic data capture tools) was noted in 9 countries. This was mainly linked to the inability to access operational funds.

## Discussion

The COVID-19 vaccination coverage rates in the countries in this study indicate that the majority have not yet attained 10% coverage with more than half at levels below 5% as of the end of December 2021. The coverage rates contrast sharply with coverage attained in developed countries in 2021 [11].

The first year of COVID-19 vaccination was characterised by the shortage of vaccines for most countries in the African Region. The global supply of vaccines started to show some improvement in August 2021. Equally important was the lack of predictability of vaccine supplies. Multiple small size shipments arriving with erratic schedules did not help countries to plan appropriately for large scale interventions. This also multiplies the administrative and logistical procedures to receive, clear and distribute vaccines. The successful deployment of COVID-19 vaccination in Israel and Bhutan is partly due to their small size and timely acquisition of large number of doses to cover a significant proportion of their population [12,13].

Some new COVID vaccines have product characteristics (dosage, packaging, temperature stability, expiry timelines, etc) different from existing program vaccines and therefore require alternative refrigeration, handling and delivery approaches. The short shelf-life of COVID-19 vaccines was a major challenge for many countries, putting pressure on program managers to distribute and implement vaccination activities without taking time to do the necessary elaborate and cascading preparations that go into such kind of decentralised mass vaccination efforts.

Shortage of operational funding was a major finding reported from 18 countries in our study. The introduction of new vaccines and the conduct of mass vaccination campaigns normally require intensive preparations, which take time and require resources. To attain high quality coverage, there are many critical preparatory steps that cannot be missed [14-17]. Guignard *et al*. argue that the introduction of new vaccines require substantial additional planning and organizational complexity to ensure optimal vaccine delivery, posing a wide range of challenges to often overstretched and underfunded health care systems, across all components of the vaccine supply chain [18].

The fact that most of the national immunisation programs could not readily access funding to cover the costs for COVID-19 vaccine introduction has undermined key activities like training, microplanning, supervision and transportation and limited the number of vaccination teams and service delivery sites. For optimal outcomes, health worker training should be practical as much as possible, with drills and simulation exercises, which have not been adequately explored among these countries. In addition to gaps in funding, the online mode of training due to the COVID restrictions may have posed limitations, unlike in other previous campaigns [16].

A World Bank report based on the COVID vaccination readiness from March 2021 concluded that a country´s ability to roll out adult vaccination programs does not necessarily depend on the strength of childhood immunisation programs [19]. The readiness assessment was done when countries had no information about the type and volume of vaccines available to them. As indicated in the IAR reports, in such contexts, countries faced difficulties in refining their deployment plans, and launching high quality preparedness activities like microplanning, training and public mobilisation. The IAR reports have also shown that the planning, service delivery and safety monitoring of COVID-19 vaccination was not done by a parallel system, but using the program experiences as well as the logistics and human resources already available within the national immunisation programs. The findings confirm that most of these countries needed to leverage on their existing strengths for this vaccine introduction [20].

The reviews have also highlighted the need for better integration of COVID-19 vaccination with other interventions, especially with routine services. This is even more important from the perspective of optimising resources, and minimising missed opportunities for vaccinating eligible persons. The integration of COVID vaccination with other health service activities like family planning and ART treatment clinics, has been well received and is running well in Zambia. These experiences need to be reviewed and documented well to help other countries learn the lessons. In the context of limited resources, careful planning and adequate preparation are critical for integration to work optimally. Careful selection of the service delivery platform, consideration of the nature of interventions, the target groups, as well as operational issues like coordination, training and supervision are all critical when considering the integration of interventions [21].

Vaccination service delivery in developing countries is challenged by poor access to preventive health care because of lack of infrastructure, limited resources and an overstretched health workforce, among others [22]. In order to address these challenges, tailored delivery strategies are required targeting communities experiencing physical or social barriers to accessing vaccination services. In this study, despite the limitation of funding resources, mobile service delivery was implemented in 11 of the 21 countries to complement fixed and outreach services.

The expansion to the target population done by a number of countries beyond the initial prioritisation in their national deployment plans was due to low uptake among the priority populations, and the short shelf-lives of the vaccine doses. At least 9 countries in the European Union implemented suspensions, delays and adjustments to the timelines by June 2021, due to various reasons including reported adverse events [23].

The perception of COVID-19 risks among the public directly affects the demand for vaccines. The low number of COVID-19 case reports in between the well-publicised waves of transmission may have convinced people to take a wait-and-watch approach. The authors´ observations and analysis of trends of vaccination in countries in the African Region shows that the uptake of vaccines has more or less mirrored the waves of COVID transmission across countries, especially with the emergence of new variants. In addition, the relatively low mortality from COVID in the majority of African countries, as compared to other parts of the world, is likely to be an important factor for people´s decisions whether to get vaccinated or not.

Vaccine hesitancy refers to a delay in acceptance or refusal of vaccination despite availability of vaccination services [24]. The rapid pace of development of COVID-19 vaccines and the development of novel mRNA vaccines were the subjects of misinformation and dis-information on social media, contributing to hesitancy [25,26]. Communication on the topic of COVID-19 vaccination is also compounded by the different types of vaccines, with different dosing schedules, and stockouts that prevented timely provision of second dose vaccines.

In our study, a number of countries have documented some type of vaccine hesitancy related to various factors. Misinformation was identified as a major challenge. There was some product-specific hesitancy targeting AstraZeneca vaccine in many countries in the African region following the widely publicised reports of thrombotic adverse events after AstraZeneca vaccination [27]. Eswatini experienced similar population reluctance following AEFIs cases. Burke *et al*. argue that trust in vaccine approval, the perceived effectiveness of the vaccine for protecting others, and conspiracy beliefs are the most significant drivers of intentions to vaccinate [28]. The Netherlands reported that some drivers of low vaccination acceptance and uptake in some groups include low perception of risk, doubt about long-term effects/side effects, lack of trust in government in general, misinformation or not enough information [29].

Studies on public attitudes to COVID-19 vaccination in various African countries show important gaps along with differences in acceptance rates between urban and rural areas [30]. An exception in this study was the almost universal acceptance documented in Ethiopia. However, another study in Addis Ababa showed a comparatively higher likelihood of hesitancy among females and persons who depend on social media as their primary source of information [31]. Similar findings were reported from Turkey and Egypt [32,33]. In a community based study in the province of Tamil Nadu in India, it was noted that younger persons, women and rural residents, and persons of low income had higher levels of hesitancy to COVID vaccines [34]. On the other hand, studies from Saudi Arabia and Pakistan showed better knowledge and acceptance rates [35,36]. In Israel, the mass media helped create public assurance by providing the number of persons vaccinated on a daily basis and showing long queues for vaccination services [12].

During the IARs, at least 5 countries have documented hesitancy among health care workers. Similar findings have been documented in studies published from other countries [37-40]. Hesitancy among health workers is partly linked to training gaps and to inadequate efforts to understand and address their misconceptions. Countries should prioritise studies focused on documenting the specific knowledge gaps and attitudes of health workers and use the information to appropriately address the needs. However, most of these studies were done in the early months of COVID vaccination and may not reflect later attitudes. As more and more people get vaccinated, public attitudes are likely to change positively, with the observation that the feared safety concerns with COVID vaccination are actually not true. Following a nearly one-year period of service delivery in many countries, there is a need for more up-to-date knowledge of public perceptions through rapid field studies to better understand attitudes, tailor messaging and design appropriate interventions.

Countries in the African Region have adequate experience with mass vaccination campaigns over the last two decades [16]. With ample preparation and adequate operational funding, there is a high likelihood of attaining boosts in vaccination coverage getting countries nearer to their targets. COVID-19 will likely continue to circulate for some time, and vaccination with primary and booster doses will need to continue until critical levels of population immunity are attained.

The IAR reports identified a number of new practices including the use of digital platforms for the pre-registration of clients, the use of mobile tools for the capture of data on reported AEFIs; the use of cold chain temperature monitoring tools while moving vaccines to subnational depots, the use of different software platforms for real-time capture of service monitoring data. The ultimate lesson to be learnt is the need for countries to make better investments in terms of establishing emergency preparedness and allocate adequate emergency resources to mobilise when a crisis arises [12,41].

This study has some limitations. The findings from these 22 countries do not represent the situation across the whole African Region, and are specific to the time and the context in the weeks and months prior to the IAR exercises. Moreover, the challenges and practices reported here are highly contextual and may be subnational in nature and not necessarily reflect the situation nationwide. The depth of documentation in the IAR reports was not uniform, and the reports may not capture all the key elements that came out of the review exercise. All except the Kenya study were done as desk exercises. The timing of the IAR exercise vis-à-vis the vaccine supply situation also determines the findings. Most of the COVID-19 vaccination IAR exercises were conducted in the months of low vaccine supply, and the experience with COVID vaccination was only for a few months by the time of the IAR. Most of the exercises were done as stand-alone reviews focusing on the vaccination pillar of the response, and tend to have adequate details on the vaccine. A few of the IAR exercises were done as part of an intra-action review focusing on the whole COVID-19 response effort, thus may be lacking the necessary granularity on the vaccination response.

## Conclusion

Intra-action reviews are useful tools in identifying strengths and weaknesses of ongoing COVID-19 vaccination activities. WHO recommends that countries conduct IARs regularly, as this enables evaluation of critical challenges and good practices. In addition, the recognition of innovative ideas and practices is important from the perspective of motivating the workforce. The roll-out of COVID vaccination presents various challenges that can be addressed through the mobilisation of political will and resources, as well as careful preparation. The IARs present perfect opportunities to identify and address program gaps. In addition, COVID-19 vaccination also presents opportunities for innovations in various areas of program performance that require careful documentation and dissemination for possible scaling-up.

### What is known about this topic

- Various COVID-19 vaccines have been developed and have been put to use within a year of the start of the pandemic - Hesitancy towards COVID-19 vaccines has been documented in many countries;
- Mass vaccination exercises require clearly committed and quantified resources, careful planning and adequate preparation;
- Intra-action reviews are widely used in emergency contexts to document early lessons and implement the necessary adjustments to the implementation of interventions, similar to post-introduction evaluations in the case of new vaccine introductions.

### What this study adds

- Despite strong high level political commitment at national level, COVID-19 vaccination in most countries was heavily challenged by the erratic supply of vaccines and a shortage of operational funds. Funding gaps prevented countries from putting in place the required intensive preparations, and the short shelf-life of the vaccines led to rushed implementation;
- Countries identified gaps in health workers training and supervision, as well as in AEFI reporting and safety monitoring - countries have gained valuable experiences exploring various COVID-19 vaccination service delivery models, including implementing the integration of COVID vaccination within routine health care programs;
- There is a need to regularly monitor or do studies measuring public perceptions towards COVID-19 vaccination in order to drive the communication and demand generation efforts, as well as use evidence in addressing hesitancy.
